# Incidence of and Risk Factors Associated with Pulmonary and Extra-Pulmonary Tuberculosis in Saudi Arabia (2010–2011)

**DOI:** 10.1371/journal.pone.0095654

**Published:** 2014-05-13

**Authors:** Ziad Ahmed Memish, Elija Afolabi Bamgboye, Naila Abuljadayel, Hanan Smadi, Mohamed Salamh Abouzeid, Rafat Faisal Al Hakeem

**Affiliations:** 1 Public Health Directorate, Ministry of Health, Riyadh, Kingdom of Saudi Arabia; 2 College of Medicine, Alfaisal University, Riyadh, Kingdom of Saudi Arabia; 3 Department of Epidemiology and Medical Statistics, University of Ibadan, Ibadan, Nigeria; Colorado State University, United States of America

## Abstract

**Setting:**

National Tuberculosis Program, Department of Public Health, Ministry of Health, Kingdom of Saudi Arabia (KSA).

**Objective:**

To summarize data on the incidence of tuberculosis and associated risk factors for cases reported during 2010–2011.

**Design:**

Retrospective analysis of routinely collected data through an established national disease notification system of the Ministry of Health in KSA.

**Results:**

The estimated incidence of all forms of tuberculosis fell from 15.8/100000 (95% CI: 15.3/100,000–16.3/100,000) in 2010 to 13.8/100,000 (95% CI: 13.4/100,000–14.2/100,000) in 2011. Saudis experienced a decrease from 11.8/100,000 (95% CI: 11.3/100,000 to 12.3/100,000) in 2010 to 9.9/100,000 (95% CI: 9.5/100,000–10.4/100,000) in 2011 while the incidence in non-Saudis declined from 24.7/100,000 (95% CI: 23.6/100,000 to 25.7/100,000) in 2010 to 22.5/100,000 (95% CI: 21.5/100,000 to 23.4/100,000) in 2011. The proportion of Extra Pulmonary TB (EPTB) which increased minimally from 30% in 2010 to 32% in 2011 was higher than global figures and strongly associated with age, sex, nationality and occupation.

**Conclusion:**

The current estimated incidence of about 14/100,000 in 2011 is less than half its estimated value of 44/100000 in 1990. Without prejudice to any under-reporting, the KSA appeared to be on the course for TB elimination by 2050 having reached the first milestone set by WHO. The proportion of EPTB remains higher than global figure and age, sex, nationality and occupation were significant independent predictors of EPTB.

## Introduction

With an estimated 8.5–9.2 million cases and 1.2–1.5 million deaths in 2010, tuberculosis (TB) is the second leading cause of death after HIV from any single agent and continues to be a major global public health concern. [1] The target set by the STOP TB Partnership, WHO and Millennium Development Goals is to reduce the prevalence of TB to half of its value in 1990 by 2015. KSA has made tremendous efforts towards the world plan to eliminate TB. The incidence of new TB cases in KSA of 22/100,000 in 2009 and this compared favorably with the situation in developed countries, and much lower than the overall incidence of 174/100,000 reported in the WHO EMRO region in the same year is noteworthy. [1]–[3] Interestingly, the WHO has signified of its intention to make KSA a model country in the worldwide elimination of TB. [4] Therefore, the current study examines the reported cases of TB in 2010 and 2011 with special interest in its epidemiology that may assist the National Tuberculosis Program (NTP) in KSA revise their policies to achieve their goal of elimination.

## Methodology

KSA has a well-established national infectious diseases notification system whereby all the different health affairs (13 provinces and 7 districts) and report all notifiable diseases to the central registry at the Ministry of Health. It is mandatory to notify the health authority of any case of TB diagnosed from any of the health institutions to the Regional Directorate of Health Affairs. The data on TB for 2010 to 2011 for new cases were retrieved and analyzed retrospectively using SPSS (version 15.0). [5] Data for the denominator of the incidence rate (population of KSA) were derived from Saudi Arabia Statistical Health Statistics 2010 and 2011. The binary logistic regression model was fitted to the data with type of TB as dependent variable and age, sex, nationality and occupation as independent variables. This binary logistic analysis technique was used to assess the association between risk factors (predictors) and the probability of TB patient being pulmonary (PTB) or extra-pulmonary (EPTB) at 5% significance level. The forward selection procedure was used to fit the variables and the results of the Hosmer-Lesmeshow tests revealed that the model fit was adequate. [5].

## Results

### Incidence Rate of Tuberculosis

Table 1 showed 70% of the 4,293 new TB patients notified in 2010 as well as the 3,872 cases in 2011 (a 10% decline) were PTB and the remaining 30%, EPTB. The commonest diagnosis of EPTB were lymphadenitis (45%), central nervous system (7%), gastrointestinal (5%), and renal TB (0.7%) while about 31% were not defined with respect to site of disease as presented in Table 2. Given that the total population of the KSA in 2010 was 27,136,977, the overall estimated incidence rate of TB was 15.8/100,000 (95% CI: 15.3/100,000–16.3/100,000). The estimated population of Saudi Arabia in 2011 was 28,016,602 at a growth rate of 3.19% per annum. Thus the estimated incidence of TB in 2011 was 13.8 per 100,000 population (95% CI: 13.4/100,000–14.2/100,000) a decline of about 13% from the rate in 2010.

**Table 1:** The distribution of new TB cases by Pulmonary and Extra-Pulmonary among Saudis and Non-Saudis (2010–2011).

Nationality	2010		Total 2010	2011		Total 2011
	Pulmonary (%)	Extra-Pulmonary		Pulmonary (%)	Extra-Pulmonary	
** Non-Saudi Arabia**	1503(72.3)	575(27.7)	2078(48.4)	1366(69.8)	591(30.2)	1957(50.5)
** Saudi**	1485(67.0)	730(33.0)	2215(51.6)	1275(66.6)	640(33.4)	1915(49.5)
**TOTAL**	2988(69.6)	1305(30.4)	4293(100.0)	2641(68.2)	1231(31.8)	3872(100)

**Table 2:** The sites of new Extra-Pulmonary Tuberculosis patients in Saudi Arabia by nationality 2010–2011.

Type	2010		Total	2011		Total
	Saudi	Non-Saudi		Saudi	Non-Saudi	
BONE	64 (8.8)	36 (6.3)	100 (7.7)	74(11.6)	35 (5.9)	109 (8.9)
GASTROINTESTINAL	46 (6.3)	21 (3.7)	67(5.1)	41 (6.4)	29 (4.9)	70 (5.7)
LYMPHATIC	320(43.8)	270(47.0)	590(45.2)	278(43.4)	260(44.0)	538(43.7)
MILIARY	19 (2.6)	13 (2.3)	32 (2.5)	15 (2.3)	15 (2.5)	30 (2.4)
NERVOUS	57(7.8)	40 (7.0)	97 (7.4)	45 (7.0)	39 (6.6)	84 (6.8)
RENAL	6(0.8)	3 (0.5)	9 (0.7)	3 (0.5)	4 (0.7)	7 (0.6)
OTHERS	218(29.9)	192(33.4)	410(31.4)	184(28.8)	209(35.4)	393(31.9)
**TOTAL**	**730**	**575**	**1305**	**640**	**591**	**1231**

The non-Saudis in 2010 with a population of 8,429,401 constituted 31.06% of the total population of the KSA. And the incidence rates of TB estimated separately for Saudis and non-Saudis were 11.8/100,000 (95%CI: 11.31/100,000 to 12.29/100,000) and 24.7/100,000 (95%CI: 23.6/100,000 to 25.7/100,000) respectively. The difference in the incidence rates between the Saudis and non- Saudis was statistically significant, P<0.001. The incidence rates declined in 2011 to give 9.92/100,000 (95% CI: 9.48/100,000–10.36/100,000) for Saudis and 22.49/100,000 (95% CI: 21.49/100,000 to 23.4/100,000) for non-Saudis respectively.

### Smear Analysis

The proportion of all new TB patients in 2010 and 2911 that were smear-positive was higher among non-Saudis than the Saudis P<0.05. However, a lower proportion was smear negative in 2010, lower among non-Saudis (11%) compared to Saudis (15%). The proportion of EPTB patients slightly increased from 30% in 2010 to 32% in 2011. The increase was more noticeable among the non-Saudis than the Saudis as shown in Table 3. In each year, about 13% of PTB patients were smear negative while about 2% of the patients did not have result of smear analysis.

**Table 3:** The distribution of smear positives, negative and EPTB in new tuberculosis patients in Saudi Arabia by nationality and year.

Smear Analysis	2010		Total	2011		Total
	Saudi	Non-Saudi		Saudi	Non-Saudi	
Positive	1079(48.7)	1222(58.8)	2301(53.6)	913(47.7)	1142(58.3)	2055(53.1)
Negative	332(15.0)	237(11.4)	569(13.3)	319(16.7)	206(10.5)	525(13.6)
EPTB	730(33.0)	575(27.7)	1305(30.4)	640(33.4)	591(30.2)	1231(31.8)
Not done	77(3.5)	46(2.7)	118(2.7)	44(2.2)	21(1.1)	65(1.7)
**TOTAL**	**2215**	**2078**	**4293**	**1915**	**1957**	**3872**

### HIV Prevalence among New TB Patients

Table 4, Panel 1 showed a total of 77 and 66 new TB patients were also HIV positive in 2010 and 2011 respectively, giving a prevalence of 1.8% (95% CI: 1.4%–2.2% and 1.7% (95% CI: 1.3%–2.1%) respectively among new TB patients. The TB patients from the Africanregion has the highest proportion of HIV positives with an overall prevalence of 8% among new TB patients followed by patients from the Eastern Mediterranean region (WHO EMRO) which accounted for about 24% giving a prevalence of 2.5% over the two years of study but slightly higher in 2010. There were no HIV patients from countries in the European region and neither TB nor HIV were from the Americas and 1.2% of the new TB patients who are Saudi Arabia citizens were positive for HIV increasing from 1.1% in 2010 to 1.4% in 2011.

**Table 4:** The distribution of new tuberculosis patients in Saudi Arabia Province/District (Health Affairs) by type 2010–2011.

District	2010		Total	2011		Total
	Pulmonary	Extra-Pulmonary		Pulmonary	Extra-Pulmonary	
**Aseer**	97 (3.0)	52 (3.9)	149 (3.3)	83 (3.0)	51(4.1)	134 (3.3)
**Albaha**	29 (0.9)	6 (0.4)	35 (0.8)	30 (1.1)	17(1.4)	47 (1.2)
**Bishan**	14 (0.4)	7 (0.5)	21(0.5)	8 (0.3)	5 (0.4)	13 (0.3)
**Eastern**	159 (5.0)	40 (3.0)	199 (4.4)	191 (6.8)	21 (1.7)	212 (5.7)
**Hafr El-Baten**	28 (0.9)	18 (1.3)	46 (1.0)	22 (0.8)	8 (0.6)	30 (0.7)
**Hail**	16 (0.5)	8 (0.6)	24 (0.5)	13 (0.5)	5 (0.4)	18 (0.4)
**Hassa**	71 (2.2)	79 (5.9)	150 (3.3)	42 (1.5)	60 (4.8)	102 (2.5)
**Jizan**	219 (6.8)	59 (4.4)	278 (6.1)	179 (6.4)	56 (4.5)	235 (5.8)
**Jeddah**	978(30.5)	257 (19.2)	1235 (27.2)	883 (31.5)	303(24.3)	1186 (29.3)
**Al-Joof**	16 (0.5)	22 (1.6)	38 (0.8)	14 (0.5)	6 (0.5)	20 (0.5)
**Medinah**	149 (4.6)	42 (3.1)	191 (4.2)	150 (5.4)	49 (3.9)	199 (4.9)
**Makkah**	243 (7.6)	43 (3.2)	286 (6.3)	179 (6.4)	40 (3.2)	219 (5.4)
**Najran**	25 (0.8)	27 (2.0)	52 (1.1)	33 (1.2)	23 (1.8)	56 (1.4)
**Nothern**	18 (0.6)	9 (0.7)	27 (0.6)	13 (0.5)	15 (1.2)	28 (0.7)
**Qaseem**	81 (2.5)	58 (4.3)	139 (3.1)	100 (3.6)	43 (3.4)	143 (3.5)
**Qounfoda**	24 (0.7)	10 (0.7)	34 (0.7)	23 (0.8)	8 (0.6)	31 (0.8)
**Qurayat**	6 (0.2)	5 (0.4)	11 (0.2)	7 (0.2)	0 (0.0)	7 (0.2)
**Riyadh**	912(28.4)	541 (40.4)	1453 (32.0)	700 (25.0)	489 (39.2)	1189 (29.3)
**Tabouk**	49 (1.5)	26 (1.9)	75 (1.6)	55 (2.0)	20 (1.6)	75 (1.9)
**Taif**	74 (2.3)	30 (2.2)	104 (2.3)	78 (2.8)	30 (2.4)	108 (2.7)

The proportion of new TB cases positive for HIV were higher in Jeddah (3.8%) and Asser (3.2%) than the national average (1.8%) and other health regions of Saudi Arabia.

### Province/District (Health Affairs)

The distribution of the new TB cases according to the Health Affairs (13 provinces and 7 districts) classification within the KSA is shown in Table 4. More than 60% of the TB patients were from Jeddah and Riyadh and another 15% from the Eastern health affair and holy Mecca. The remaining 25% were located in the remaining nine health affairs of the Kingdom with Jazan contributing about 7%. Also, about a third of non-Saudi TB patients were from Jeddah, higher than the 23% of Saudis but an almost equal proportion (30%) of Saudi and Non- Saudi were from Riyadh. A higher proportion (77% and above) of TB patients from Mecca, Jeddah, Jazan, and Eastern health affair were of the pulmonary type, much higher than the proportion of patients from Riyadh (62%). Riyadh, the capital of KSA which contributed about 32% of all new TB patients in 2010 accounted for more than 40% of EPTB. This pattern was sustained in 2011 even though a majority of the health affairs reported lesser cases. The Eastern health affair appeared to be the notable area with an increased frequency of new TB patients in 2011.

### Risk Factors Associated with Extra-pulmonary Tuberculosis

The overall mean age of patients with PTB (36.4 years, SD = 17.0 years) appeared similar to patients with EPTB (35.0 years, SD = 16.4 years) as shown in Table 5. But the difference was statistically significant, P<0.05. The mean age of 35.6 years, (SD = 16.8 years) for patients in 2010 was also statistically significantly lower than the mean of 36.4 years (SD = 16.8 years) for patients in 2011 (P<0.05).

**Table 5:** The distribution of new tuberculosis patients in Saudi Arabia by type and selected demographic characteristics 2010–2011.

		2010		Total 2010	2011		Total 2011
		Pulmonary (%)	Extra-Pulmonary		Pulmonary (%)	Extra-Pulmonary	
**Age group**	0–4	15(0.5)	24(1.8)	39(0.9)	10(0.4)	20(1.6)	30(0.8)
	5–14	80(2.7)	62(4.8)	142(3.3)	60(2.3)	52(4.2)	112(2.9)
	15–24	731(24.5)	249(19.1)	980(22.8)	561(21.2)	233(18.9)	794(20.5)
	25–34	923(30.9)	421(32.3)	1344(31.3)	804(30.4)	420(34.1)	1224(31.6)
	35–44	426(14.3)	244(18.7)	670(15.6)	435(16.5)	204(16.6)	639(16.5)
	45–54	348(11.6)	136(10.4)	484(11.3)	344(13.0)	124(10.1)	468(12.1)
	≥65	242(8.1)	91(7.0)	333(7.8)	221(8.4)	87(7.1)	308(8.0)
**Sex**	Female	1113(37.2)	567(43.4)	1680(39.1)	1006(38.1)	525(42.6)	1531(39.5)
	Male	1875(62.8)	738(56.6)	2613(60.9)	1635(61.9)	706(57.4)	2341(60.5)
**Occupation**	Skilled workers	174(5.8)	74(5.7)	248(5.8)	172(6.5)	72(5.8)	244(6.3)
	Semi-skilled (Vocational training)	222(7.4)	73(5.6)	295(6.9)	258(9.8)	70(5.7)	328(8.5)
	Unskilled workers	365(12,2)	132(10.1)	497(11.6)	355(13.4)	179(14.5)	534(13.8)
	Housewives and workers	882(29.5)	454(34.8)	1336(31.1)	789(29.9)	405(32.9)	1194(30.8)
	Self-employed	1107(37.0)	464(35.6)	1571(35.6)	844(32.0)	381(31.0)	1225(31.6)
	Students	238(8.0)	108(8.3)	346(8.1)	223(8.4)	124(10.1)	347(9.0)
**Nationality**	Eastern Mediterranean	508(17.0)	158(12.1)	666(15.5)	484(18.3)	199(16.2)	683(17.6)
**(WHO Regions)**	South East Asia(SEAR)	572(19.1)	302(23.1)	874(20.4)	508(19.2)	256(20.8)	764(19.7)
	Western Pacific Region(WPR)	109(3.6)	47(3.6)	156(3.6)	127(4.8)	58(4.7)	185(4.8)
	African Region(AR)	286(9.6)	59(4.5)	345(8.0)	222(8.4)	72(5.8)	294(7.6)
	European Region(EUR)	19(0.6)	8(0.6)	27(0.6)	20(0.8)	2(0.2)	22(0.6)
	Others not Indicated	9(0.3)	1(0.1)	10(0.2)	5(0.2)	4(0.3)	9(0.2)
	The Americas	0	0	0	0	0	0
	Saudi Arabia	1485(49.7)	730(55.9)	2215(51.6)	1275(48.3)	640(52.0)	1915(49.5)
	Western Pacific Region(WPR)	109(3.6)	47(3.6)	156(3.6)	127(4.8)	58(4.7)	185(4.8)
**TOTAL**		**2988**	**1305**	**4293**	**2641**	**1231**	**3872**

The proportion of female EPTB patients was higher than their PTB counterparts, P<0.01. The association between patient occupation and type of TB was also statistically significant, P<0.01. Housewives and workers sharing the same household environment had more proportions with EPTB than those who were self-employed. About 71% of the non-Saudis had pulmonary TB, slightly higher than their Saudi counterparts P<0.01.

The odds ratios for each of the variables age, sex, nationality and occupation and their 95% confidence limits from the logistic regression analysis presented in Table 6 revealed them as significant predictors of the probability of a TB patient being of the Extra Pulmonary type P<0.01. Younger TB patients were more likely to be EPTB than older patients. For example a TB patient less than 5 years of age was almost 5 times more likely to have EPTB than was a patient 65 years and above. Similarly, females and non-Saudis were more likely to be diagnosed with EPTB. Also, TB patients with semi-skilled jobs were statistically significantly less likely to have EPTB compared to students which was the reference category.

**Table 6:** Logistic regression predicting extra-pulmonary tuberculosis from occupation, age, nationality, and sex.

Variable		Odds ratio	95% CI OR	P value
**Occupation**	Skilled	0.96	0.72–1.27	0.748
	Semi-skilled	0.73	0.55–0.98	0.034
	Unskilled	1.13	0.87–1.47	0.349
	House-jobs	1.02	0.82–1.27	0.848
	Self-employed	0.96	0.78–1.18	0.663
	Student (ref)	1		
**Age**	0–4	4.69	2.77–7.96	<0.001
	5–14	2.07	1.49–2.88	<0.001
	15–24	1.03	0.83–1.28	0.768
	25–34	1.43	1.17–1.75	<0.001
	35–44	1.55	1.25–1.92	<0.001
	45–54	1.11	0.88–1.39	0.386
	55–64	1.10	0.86–1.41	0.462
	65+(ref)	1		
**Nationality**	Non Saudi	1.33	1.19–1.49	<0.001
	Saudi(ref)	1		
**Sex**	Female	1.21	1.08–1.36	0.001
	Male(ref)	1		

## Discussion and Conclusion

The overall incidence rate of TB in Saudi Arabia observed in this study was estimated at about 16 and 14 per 100,000 populations for 2010 and 2011 respectively. According to the World Health Organization report published in 2011, [2] the incidence of TB was reported as 18 per 100,000 people in KSA. Available reports have shown that the incidence of 16 per 100,000 observed in 2010 appeared similar to what it was since 200. [3] But the decline in 2011 may be a cumulative effect of the gains in the control mechanism to eliminate TB in the Kingdom. The estimated incidence rate of TB in 1990 was 44 per 100,000 and the current estimate which has remained stable for three years is about half the incidence in 1990. If one recognizes that one of the targets set to achieve the objective of WHO for TB elimination was to reduce its incidence rate to half of its value in 1990 by 2015, one could safely conclude that Saudi Arabia is on track. Although, there could be some reservations that the TB data source in KSA might suffer from under reporting and consider effect seriously the interpretation of this study. But one should also realize that while the likelihood of under-reporting of TB cases cannot be wished away, there is also the fear of over-reporting because of lack of central system for the reporting a case. The NTP in KSA has integrated the care of TB into the health care system and all hospitals under the Ministry of Health, the special hospitals for the armed forces such as the National Guard, the military hospital, the security forces, other private hospitals, and health care centers all provide care and support for TB patients. But each of these hospitals report new TB patients directly to the Ministry of Health database, someone might receive care in one hospital where he has been registered as a new patient only to present in another hospital as a new patient. Perhaps the over reporting may offset the under reporting, but this study recommends an assessment of the completeness of registration of tuberculosis in the Kingdom in a separate study. But one may infer from this study that a significant progress in implementing the STOP strategy of TB in line with international recommendations has been made in KSA and a good evidence of a high-level political support and utilization of available resources. [4] The economic fortunes of Saudi Arabia with a stable GDP per capita around 20,300 dollars over the years and remarkable investment of about 7% of her annual budget usually allocated to health care services. [6] This is expected to facilitate the dream of eliminating TB to less than 1/100.000 by the year 2050. [7].

The observed proportion of EPTB in this study is an increase over the figures in 1997 when EPTB accounted for 28.2% of all reported TB cases [8] and the findings in 1991 when the proportion was only 11.7%. [9] The seemingly low prevalence of HIV in Saudi Arabia has defeated a link with HIV as a possible explanation. [10] It is generally known that HIV and Mycobacterium tuberculosis have a synergistic interaction and each propagates the progression of the other so that increasing HIV prevalence can show up as more cases of EPTB. [11] Therefore the increase in EPTB might probably be due to the availability of better diagnostic facilities and better reporting system, leading to the identification of more cases. Also, that the proportion of EPTB increased among non Saudis is consistent with previous reports that foreigners could change the epidemiology of TB as reported in Germany, Denmark and USA. [12]–[14].

The result of the logistic regression analysis that showed persons less than 15 years of age were likely to have EPTB than was a person 65 years and above was similar to an earlier study which reported the preponderance of EPTB in the younger age group. [9] Young age as an independent risk factor for EPTB observed in this study has also been reported in earlier studies from Nepal, USA and Europe. [15]–[17] The usual explanation has been the possibility of a higher probability of reactivation at an extra-pulmonary site at younger ages after primary infection in the lungs. That a higher proportion of PTB was found at higher ages as observed in this study may also suggest a higher likelihood of reactivation of TB in the lungs at older ages. This is more likely the effect of decreased local immunity in the lungs in the elderly. A large pool of latent tuberculosis have been reported in an earlier nationwide community study in Saudi Arabia. [18] And this is more likely to be the source of possible reactivation taken place in older ages. [19].

The significance of people in semi- skilled jobs less likely to have EPTB compared to students could also be attributed to age as the students were more likely to be younger and childhood TB is usually a reflection of new infections. That females were more likely to be diagnosed with EPTB than males was consistent with results of previous studies by Al-Otaibi [9] who reported a higher proportion of EPTB among females and others. [12], [14], [20] No clear explanation have been offered other than a suggestion of possible roles of endocrine factors.

Also the fact that non-Saudis had a significantly excess odds of EPTB compared to Saudis is not new as other studies have found that non -Saudis tended to have EPTB more than PTB. [9], [21] The pre-employment screening at entry, which prominently targets PTB could have missed out a number of EPTB which later show up for treatment. The most common site EPTB observed in this study was lymph nodes, which was similar to other previous studies from KSA, Nepal and Turkey, [15], [22], [23] which have reported that lymph nodes accounted for nearly half the cases of EPTB. Other forms of EPTB were TB of the bone, gastro and CNS which is consistent with other studies from Saudi Arabia. [1], [21] The fact that more than 60% of patients TB patients were received from Jeddah and Riyadh health affairs is not surprising because the foreigners work in the major cities of the country. Riyadh, which contributed about 32% of all new tuberculosis patients in 2010 accounted for more than 40% of EPTB. This again can be explained as a fall out that the foreigners were more likely to be based in the capital city, [6] and a study from the USA reported higher rates in cities. [24] The observed 54% smear-positive among new TB patients in this study is consistent with what was observed in KSA in previous years but higher than global values. [1], [21] However, the non-Saudis (59%) have maintained their higher values than Saudis (49%). These values were higher than values in the Eastern Mediterranean region. One cannot readily explain this differential. Also the overall proportion of smear negative (13%) which was lower among non-Saudis (11%) and higher among Saudis (15%) were generally lower than global values. [1].

The results of our study corroborate similar studies that found a decrease in the overall TB incidence. The current estimate of the incidence rate of TB of about 14/100000 is less than half its value of 44/100000 in 1990. Without prejudice to any under-reporting of cases, the KSA appeared to have reached the first milestone set by WHO for TB eradication by 2050. The proportion with EPTB among new TB patients remains higher than global figures and suggested younger age, non-Saudis, female gender and semi- skilled occupation may be its significant independent risk factors for EPTB relative to PTB in KSA. Based on our results TB control programs might target young, female, and non-Saudi populations for early diagnosis of EPTB. The Kingdom needs more attention on the role of HIV among the TB patients.
